# Designing narratives and data visuals in comic form for social influence in climate action

**DOI:** 10.3389/fpsyg.2022.893181

**Published:** 2022-09-27

**Authors:** Ray LC, Zijing Song, Yating Sun, Cheng Yang

**Affiliations:** ^1^School of Creative Media, City University of Hong Kong, Kowloon, Hong Kong SAR, China; ^2^Academy of Art University, San Francisco, CA, United States

**Keywords:** design fiction, data comics, climate action, data narratives, data visuals

## Abstract

Climate change is difficult to connect with personally, because people only regard the phenomenon as important if it becomes a perceived threat to themselves. Arguments like statistics and policy debates are extrinsic motivators, which do not necessarily align people’s own intrinsic motives with those of climate action. Instead, narratives and visual communication can influence viewers implicitly by the way they show and reinforce actions and thoughts that align with climate action. In this design study, we used comics created for human-level climate change influence to promote ideas like future-based thinking, sharing of responsibility, and caring for each other. We also created data visuals that illustrate future consequences of climate change for the purpose of averting negative alternative realities. To see whether our design can affect audience perception of climate change on the human level of goals and desires, we showed the comics to readers unfamiliar with the themes of the stories, presenting them as manga about characters and situations. The survey showed that data stories can affect the way naive readers interpret narratives to align with pro-climate attitudes such as sharing and future-vision, and that readers are focused on the human-level of the data and story as opposed to the physical resource level. Speculative fiction and data visuals provide a potentially effective way to influence individuals’ climate change attitudes by showing alternative realities and attributes of collective responsibility and planning-for-the-future as data stories.

## Introduction

Climate change is a major crisis of our generation, but public perception of it depends on factors like individual habits, socioeconomic demographics, political ideology (Weber, 2016), socio-political motivation (Luo and Zhao, 2019), psychological distance (Wang et al., 2019), personal relevance (Arıkan and Günay, 2021), and many other factors we are beginning to unravel. In particular, it can be difficult to change the mindset of even climate-conscious communities who are skeptical and apathetic about climate change. This skepticism comes from fixed attitudinal positions, skeptical voices in public media, lack of trust in decision-makers, lack of support for personal beliefs, etc. (Happer and Philo, 2016). Climate communication strategies that use more personal strategies like storytelling and visual communication, on the other hand, are suitable for developing pro-environmental behaviors and attitudes (Boykoff, 2019). These long-term behaviors for social good need to activate intrinsic motivations, which can develop when experiencing narratives aligned with particular social purposes (Lc and Mizuno, 2021). We therefore employ the genre of data comics (Bach et al., 2017), which combines speculative design of fiction (Dunne and Raby, 2013) and data visuals in the form of more-understandable and non-threatening comics to create more appealing and persuasive narratives, magazine styles, annotated story-based data charts, partitioned poster, and comic strips styles for the purposes of climate influence.

On the physical level, climate change is connected to a set of phenomena like material consumption, natural resource exploitation, increases in population, and lack of policies in sustainability. These issues depend on policy debates that are related to people ‘s pre-existing environmental attitude (Taube et al., 2021). Instead of working at the physical level, we intend to intervene at the human level, addressing phenomena like immediate gratification, human myopia, taking comfort in ignorance, belief in lack of individual influence, and desensitized negative outcomes. To promote long-term behaviors that align with climate awareness using visual communication, we use the medium of comics, which have broad appeal to capture even climate skeptic’s attention without appearing to preach about climate change. We use data communication methods embedded in the comic to diffuse the difficulty of the topic and reduce the negative reaction to scientific content, using speculative data visuals to narrate possible realities designed to provoke thinking about consequences of individual actions and alignment of intrinsic motivations with social goals for positive climate action.

In applying story design and data visuals for climate persuasion, we recognize the importance of audience perception to the design process. To help us understand (1) how story structures and visuals may improve awareness of climate issues on the level of goals and motivations, and (2) how particular data comic interpretations can potentially lead to more effective climate communication, we conducted a survey study of 60 readers to assess the type of information they reported learned from reading, providing feedback for improving the design process. Public opinion on topics of a political disposition are subject to only long-term changes (Sears and Funk, 1999), and such ingrained opinions as climate change (Howe et al., 2019) would not be expected to change with a single reading. Thus we limit our scope to illuminating a novel design strategy of narrative and visual intervention with potential ways for improving the way the public can perceive climate issues in the future to align with their own intrinsic motives.

Data comics may be more effective than other data-driven storytelling formats to make data-driven stories accessible and understandable, because the audience will be more engaging, enjoyable and comics are efficient to highlight temporal information or distributions (Wang, 2022). However, this research did not focus on studying whether data comics have the potential to create awareness and influence attitudes among audiences. In one study that used a comic book about rape victims in India as a research object, respondents believed that the comic can deliver a powerful social message to create issue-awareness among younger audiences (Chattopadhyay, 2019). Given the relative lack of studies in how climate communication based on narrative and visuals strategies are perceived by naive audiences, we aimed to investigate how people interpret climate comics designed to influence interpretation:

*RQ*1: How can speculative data comics affect audience perception in terms of telling stories that align with particular climate-positive goals or rather with education on resources and policy?

*RQ*2: What can we learn from the way people interpret data comics designed to influence pro-climate action, in order to create effective climate action communication?

## Background

### The connection between climate change attitude and risk perception

The policies, attitudes, and capabilities of various countries in supporting sustainability provides both opportunities and challenges for enacting positive environmental change. At present, worldwide interest has been devoted to energy use, the proliferation of renewable energy, and the employment of information technologies (Harrower, 2020). However, some environmentally friendly policies do not always make positive impacts. For example, carbon taxes may not have impact on consumer energy decisions, but would affect the steadiness of the financial system because of the decline in fossil fuel production and use (Ionescu, 2021a,b). Meanwhile, the pandemic inevitably increases the use of plastics, due to discarded products like disposable masks, gloves, and sanitizer bottles. Whereas a study recommends approaches like increasing the recyclability of plastics, using bioplastics, and developing new public-private partnerships, the public should also have an appropriate appreciation for the critical role the plastic sectors play (De Blasio and Fallon, 2022). Environment innovation and green finance are important drivers of sustainable development (Ionescu, 2021a,b). Thus, raising public awareness of environmental protection, and increasing green financial support for sustainable energy systems are needed for climate change mitigation.

### Design fiction as climate communication

Design fiction is a strategy for narrating potential futures by varying a particular premise (Blythe, 2017), considering potential futures within social and cultural narratives (Bleecker, 2009). Design fiction can provide a narrative strategy for social influence by showing the effects of alternative visions (Moezzi et al., 2017). Evidence suggests that narratives can strengthen the attitude-behavior relationship (Rhodes et al., 2016), so reading fiction may help activate pro-environmental behavior in individuals who already have strong pro-environmental attitudes. Research shows that climate fiction leads to greater public comprehension of negative consequences caused by climate changes compared with reading about research (Moser, 2016). Other research points out that there is no strong evidence to show climate storytelling is more persuasive than communicating evidence in real life (Jones, 2014). They do not account, however, for fiction purposely designed for *positive* persuasion for actions that can contribute to positive climate action as opposed to seeing negative effects of climate change.

### Comics and visualization in climate communication

In scientific communication, illustrations play significant roles as visual explanations (Schreiner, 1997) that reflect the structure of the concept presented (Farinella, 2018). As scholarly interest in visualization of climate change increases, climate visual imagery has been used in television, films, advertisements, and artworks for visualizing past and present climate states, and inspiring imagination about future states (O’Neill and Smith, 2014). In addition, case studies indicate that graphics can reduce climate dynamics to the human level and put climate change action on the same level as the risks people are willing to assume for a better life (Walsh, 2015). Such visual communication, reflected in the comic, has the potential to convey the complexity of reality despite being more accessible and widely available to the public (Darnhofer, 2018). Some researchers found that science comics have the potential to cultivate a continuing interest for the general public to learn science (Lin et al., 2015). Narrative transportation positively influences the effect for hero characters, which extant research demonstrates indirectly influences the persuasiveness of a story (Jones, 2014). Moreover, comics support contextual storytelling based on aspect transitions that convey mood and sense of place, allowing for implicit influence through environmental design as opposed to explicit forms of narrative influence (McCloud, 1994).

For the scope of this work, we focus exclusively on the sub-genre of science comics, broadly defined as “comics which have as one of their main aims to communicate science or to educate the reader about some non-fictional, scientific concept or theme” (Tatalovic, 2009). The scientific comic *“*may enable a wide audience of non-specialist individuals, who do not typically seek out science information, to engage with science related topics, thus focusing scientific literature” to something the public can understand intuitively (Spiegel et al., 2013).

### Speculative data visuals and data comics

Research has been directed at creating data visuals that affect viewers’ attitudes (Sheppard, 2005; Ballantyne, 2018). For example, through the analysis of two climate diagrams, “traffic light” and “planetary boundaries” (Morseletto, 2017), “understandable, meaningful and engaging” concepts in environmental science have been presented. These visuals encourage visual thinking, make data and information expressive, help viewers reconfigure thoughts, and facilitate communication with policy makers. Speculative design in this form allows for a discussion of possible future states (Dunne and Raby, 2013). Thus, viewers may reimagine thought-provoking questions through visual examination, and the data we present becomes “stories” connected to public awareness (Kim and DiSalvo, 2010). Therefore, combining the influences of design fiction and speculative data visuals may create more effective climate change communication in a narratively persuasive form.

Different genres work better for different story types. Choosing the appropriate genre depends on the complexity of the data and the intended audience. Business presentations typically use slide shows instead of comic strips, while television commercials use videos instead of flow charts (Segel and Heer, 2010). Data comics combine the merits from other visual storytelling genres that provide both the immersiveness of videos and the interactive features of infographics (Wang, 2022). Moreover, data comics utilize narrative concepts and visual information of traditional comics to express data-related insights designed to communicate complex scientific ideas (Wang et al., 2019). Thus, we developed data comics to express the narrative aspects of identifying with climate action, transferring traditional patterns of infographics presentation to narrative forms for particular social purposes.

## Methods: Narrative design

### Story and headline writing

We first specified particular design goals for climate change action. These goals are not related to physical resource arguments like overconsumption or resource destruction, but rather human psychological phenomena that we address here using persuasive influence. The phenomena we design against include issues like immediate gratification, myopic vision over the consequences of destructive action, the idea that an individual’s effect does not matter to global issues, the idea that there’s comfort in ignorance regarding scientific issues, etc. Since this research aims to reach general audiences and audiences who may be climate change skeptics, climate issues are not directly mentioned in the stories. The work may be read covertly as simple a comic rather than a form of climate fiction. The titles of the stories also reflect subtle cues such as *Redemption Park*, *New Revolia*, and *Every Flash of Light Is the Sun of Another World*, all of which evoke themes of rebirth, community responsibility, and learning from an experience.

### Comic design

We designed five stories to address different human phenomena propagating climate change. For example, one story is *Sonia McDougal*, based on the story structure of “Rebirth” and designed to dispel the idea of maximizing immediate gains over future considerations. We take the typical elements of the tragedy and turn the story plot into a more positive one in which the main character is forced to change their ways and become a better individual. The protagonists of this plot usually have redeeming qualities to show that they deserve a happy ending. Like the tragedy, the protagonist usually gets caught up in darkness and deals with the threat growing in similar stages to the tragedy plot, seeming almost non-existent at first before becoming so prominent it can no longer be ignored. However, rather than let the darkness triumph the protagonist eventually redeems themselves, negating what would otherwise be a tragic ending (Booker, 2004).

**Figure 1:**
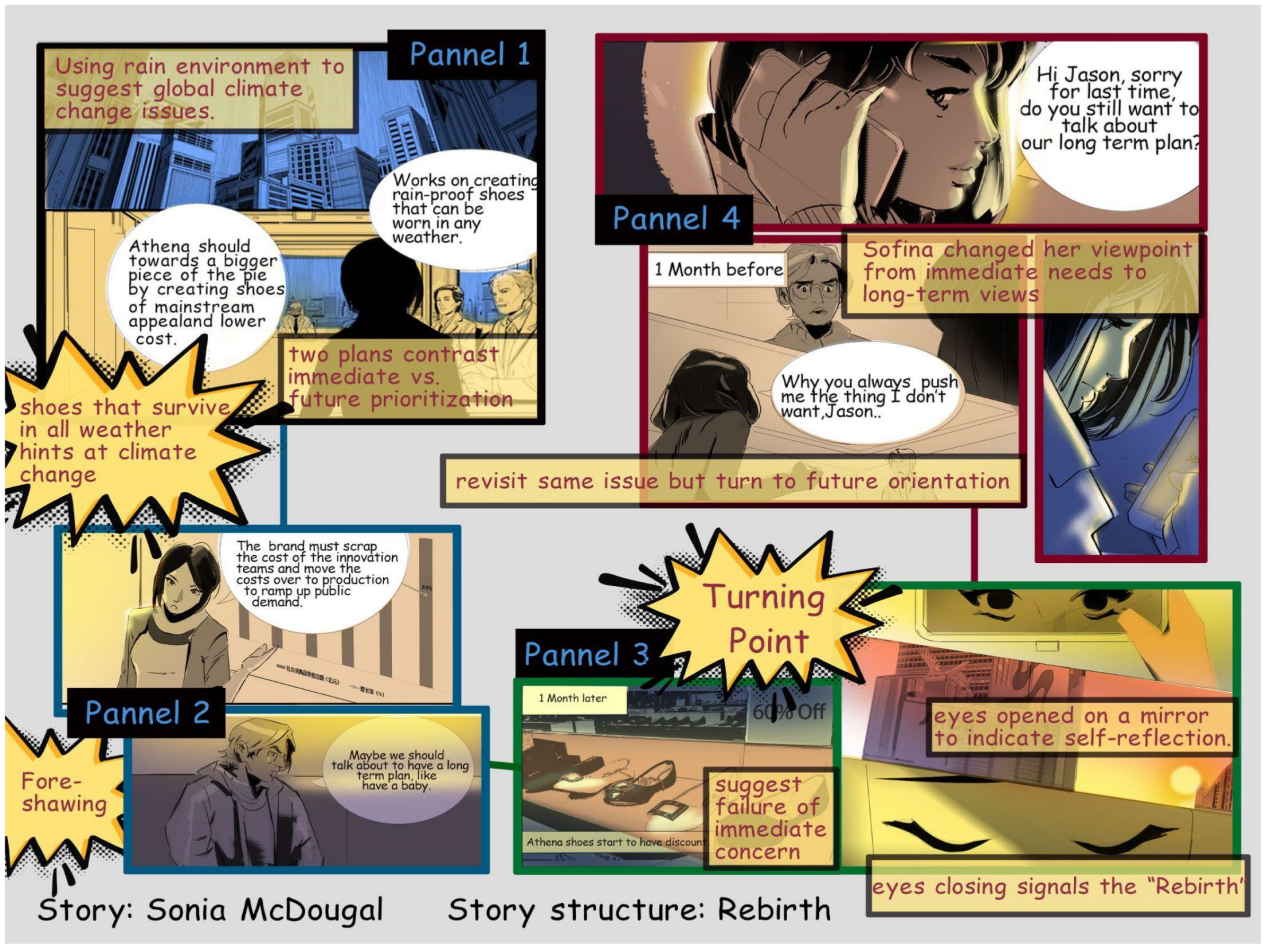
Persuasion through visual design in *Sonia McDougal*.

**Figure 2:**
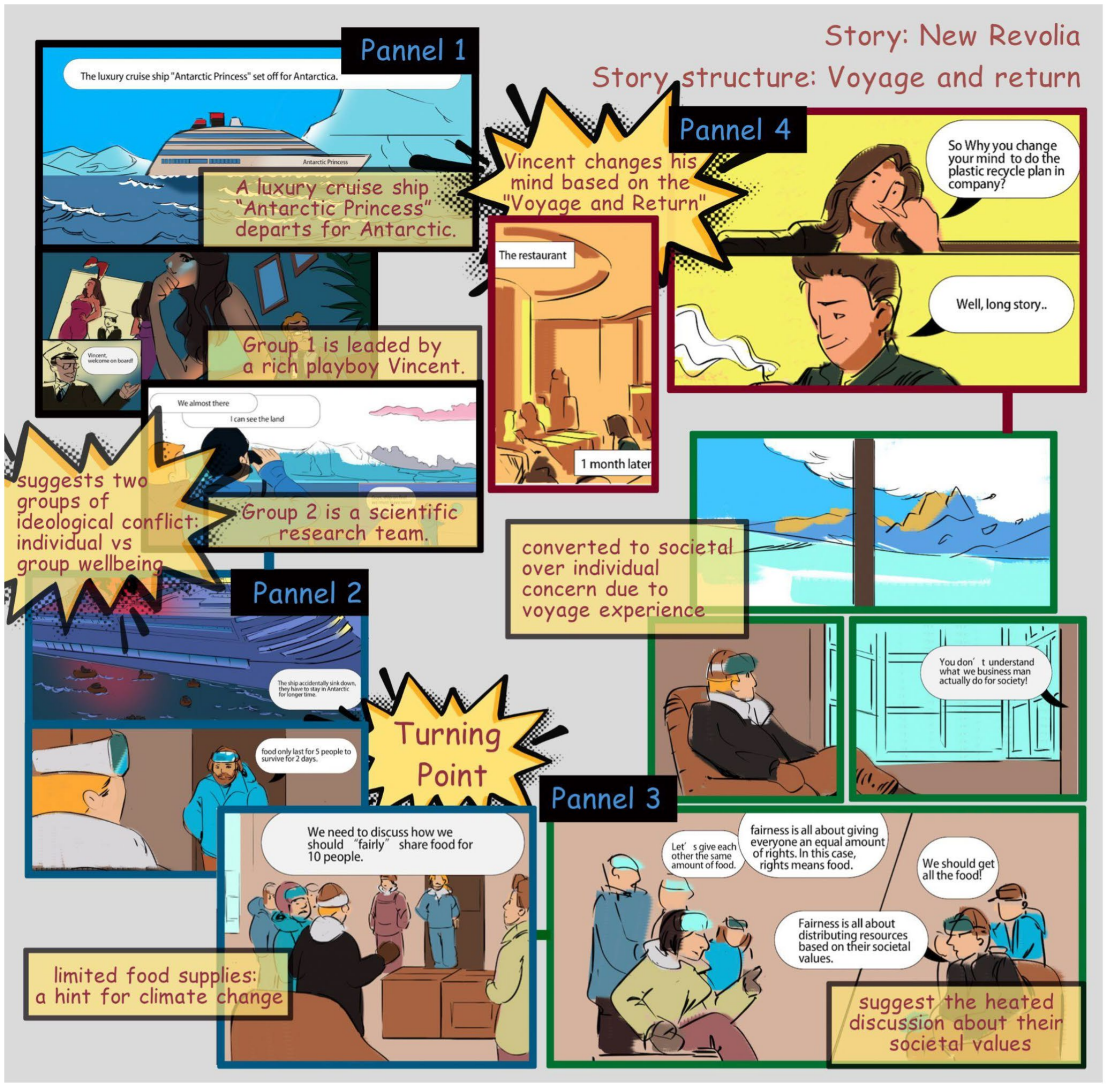
Contextual storytelling by visual design in *New Revolia*.

*Sonia McDougal* tells the story of a shoe business entrepreneur named Sonia who must make a decision about her company, whether to invest in long term research and development, or to push the product widely to the general public. In her personal life she takes the approach of immediate needs and gratification, as opposed to settling down. Then after choosing to maximize profit in her professional life, the business fails, teaching Sonia an important lesson in the “Rebirth” theme. She realizes she should consider a long-term plan and decides to take action in her personal life, to finally settle down with her boyfriend. The illustration uses a science fiction comic book style which is more to show the story theme: the yet-to-be-produced shoe which works in any weather and can predict the rain using electronics. The science fiction look subtly points out a future-directed theme in comic design, which is espousing future-directed over immediate-directed lifestyles (Figures 1, 2).

**Figure 3:**
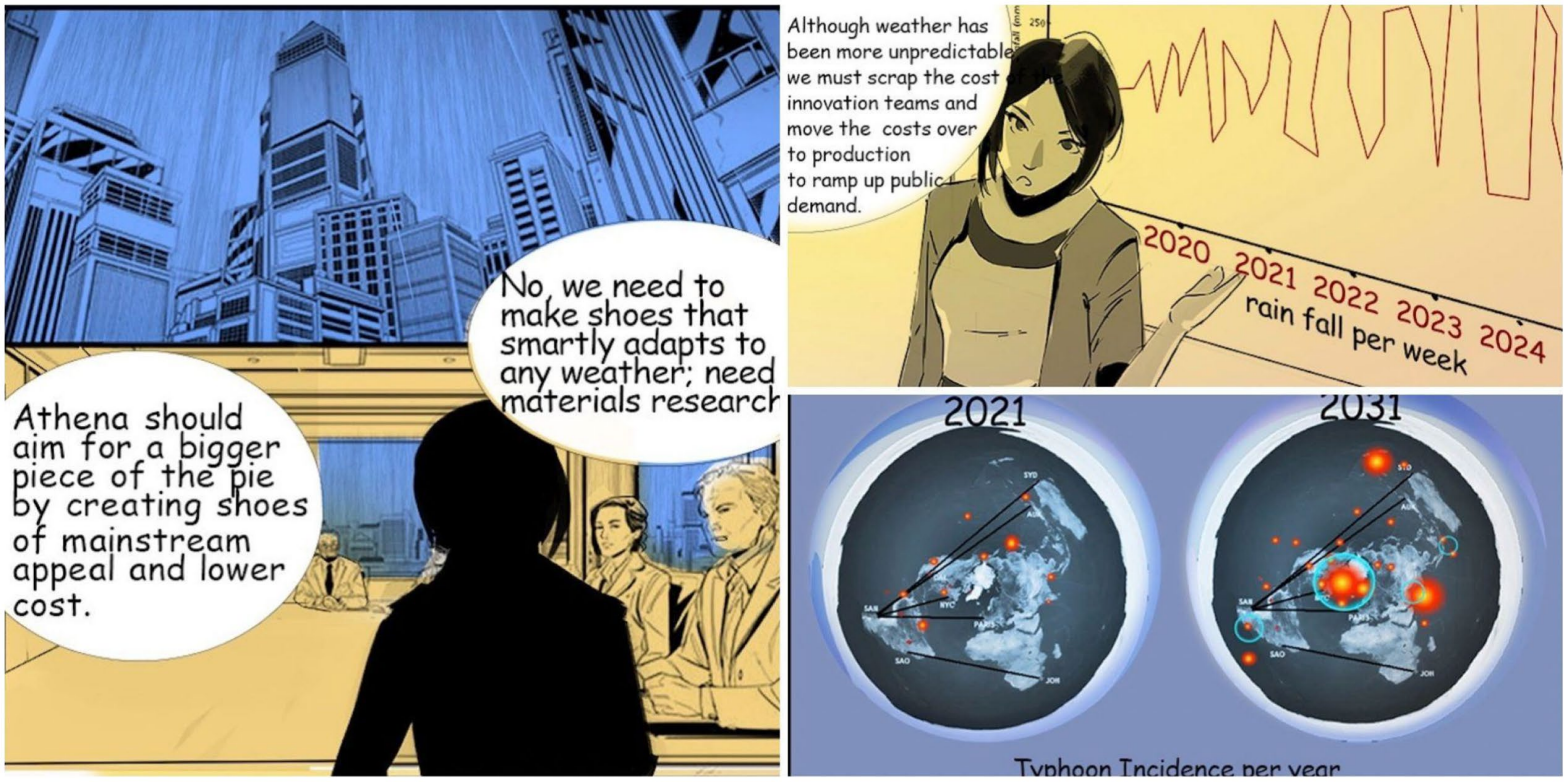
Using data visuals to describe future scenarios in the context of the narrative in *Sonia McDougal*.

Other subtle cues abound in the visual comic. For example, to show the research and development that involves experimental shoes that survive in all weather, we designed a rain motif found in several scenes in the story. To show the turning point in Sonia’s life after the business failure, we showed eyes opened on a mirror to indicate self-reflection, when she was fired from the company, and we used two panels to express the eye moment to express time flow and actually she stayed in the car awhile before leaving the company downstairs. And comics panels make the unconnected moments together, and mentally construct a continuous, unified reality. So, the audience will use closure as a grammar to understand the story. Moreover, the shoe business building is drawn in a diagonal position to indicate failing, followed by eyes closing, which signals the “Rebirth” structure that also hopes to change the audience’s own viewpoint from immediate needs to long-term views. These moments establish the change undergone by the character using aspect transitions as a way to apply environmental storytelling to the work (McCloud, 1994). The “Rebirth” theme is emphasized in the recurrent scene structures during Sonia’s two encounters with her boyfriend (Figure 3).

### Data visuals in comics

**Figure 4:**
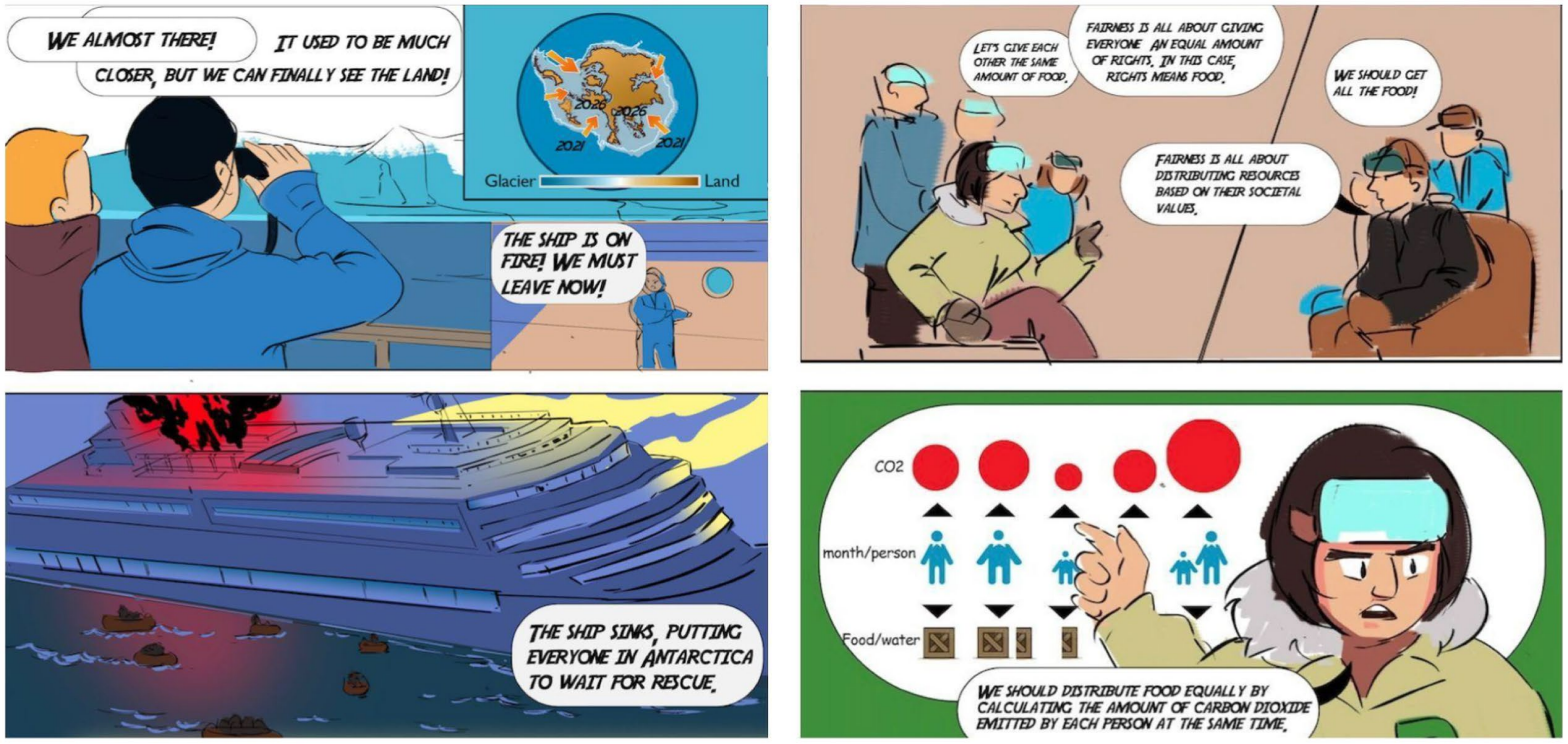
Using data visuals in *New Revolia* to describe strategies for sharing carbon emission responsibilities.

According to Bach et al. (2018), there are five major design considerations for the purposes of data comics: visualization, flow, narration, words, and pictures. In Sonia McDougal, one panel with a graph was added to show the increase in randomness of day-to-day rainfall, which is used to support the idea of research and development for future gains of the company in order to create smart rain-proof shoes (Figure 4). To keep the balance between data and context in our work while pushing forward the narrative, a distribution map on typhoon incidence in current and future times was given in the storytelling line. Note that these are not real data, but rather attempts to tell the story of future situations using data as narrative. Hence the speculative data story approach develops a hypothetical view of the world that then allows the reader to extrapolate about the consequences of following the direction of the main character in prioritizing current over future considerations.

Since comics have the advantage of splitting complex processes into less complex sections for easy understanding (Cohn, 2012), they allow viewers to follow complex relationships and scientific ideas. Two panels in New Revolia are representatives of this aspect. The first example is a graphic map that implicitly explains the main reason for the shipwreck—the melt of glaciers caused by global warming. In the turning point, a heated discussion about the social values among two groups of characters (scientists and bunnies man) occurred and one scientist put forward a reasonable solution to distribute food equally. The second example is the way the food distribution plan is visualized using graphics that not only indicate the idea of the plan, but also uses the form of the data graph in showing people and their carbon footprints to narrate the idea of sharing of responsibility in the context of the story.

### Magazine layout

**Figure 5:**
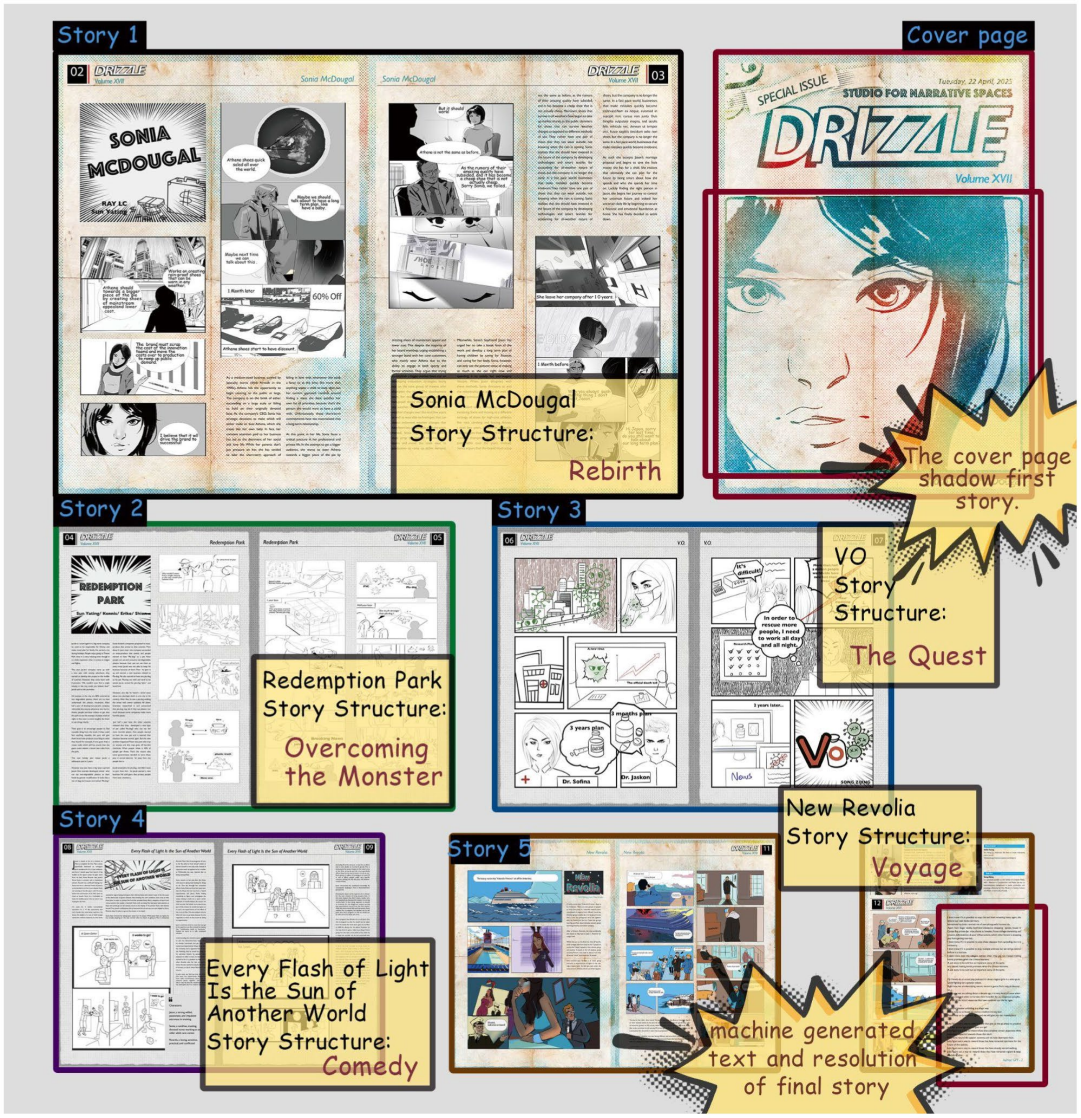
Layout of *Drizzle* and design purpose for each story.

Research has shown that what the public learns from sources like school education or newspapers makes them generally believe they have a good understanding of climate change issues (Pasquaré and Oppizzi, 2012). Thus we created the comic A4 size newspaper to mimic the style of a tabloid; A4 is widely used for magazines. The issue includes 12 pages within which are found 5 stories (*Sonia McDougal*, *Redemption Park*, *VO*, *Every Flash of Light Is the Sun of Another World*, *New Revolia*). The text fonts are custom-created to complement the comic-drawing style. In order to help readers easily understand the content and panels, the designers set the layout according to the “Z-path,” from left to right and downward, which is preferred by new comic readers (Cohn, 2013). Since the different visual emphases to page layout and panel composition have the potential to improve the dramatic effects of a story or plot, the character panels in every story are zoomed out and emphasized to help the development of storytelling (Figure 5).

## Methods: Reader study

An online survey is conducted to address RQ1, with semi-structured follow-up questions regarding story-design and perception to address RQ2 (see “Introduction”). The questionnaire was divided into demographic information, reactions to Sonia McDougal, reactions to New Revolia, and self-report of climate attitudes. First, we collected individual-level variables of participants (including age groups, gender and educational background) to be sure we get a range of different backgrounds, because some studies found that women are more concerned with environment than men and the level of education is positively related to climate change concern (Arıkan and Günay, 2021). After that, participants were asked to read the two comics and answer several questions related to their understanding and feelings about story plots, story structures, painting styles, layout, characters, data visuals and environment graphics. At this point, we ask people to interpret the comics purely on what the stories they tell and the data it visualizes, without mentioning the climate action purpose of comics. Next, we evaluate the effectiveness of the comics and how readers interpret them in context of climate change. We adapted previous work (Christensen and Knezek, 2015) for a self-efficacy scale about climate change attitude to measure how pro-climate each reader is.

**Figure 6:**
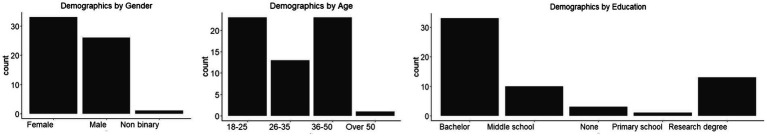
Demographic background in the survey study (N = 60, female = 33, male = 26, nonbinary = 1) broken down by Gender (left), Age Group (middle), and Education Obtained (right).

Participants were recruited and paid through the online research platform Prolific (*n* = 60, 26 males, 33 females, 1 non-binary). The demographics of the participants are shown in Figure 6. We used R 4.0.3 and RStudio 1.3 to process, analyze, and plot the data. The participants’ short responses to questions about interpretation of particular aspects of the story were then coded using open coding and independently analyzed by two evaluators. Results from the coding process are described in section 6. Participant demographics are provided in Figure 6.

## Results: Quantitative

**Figure 7:**
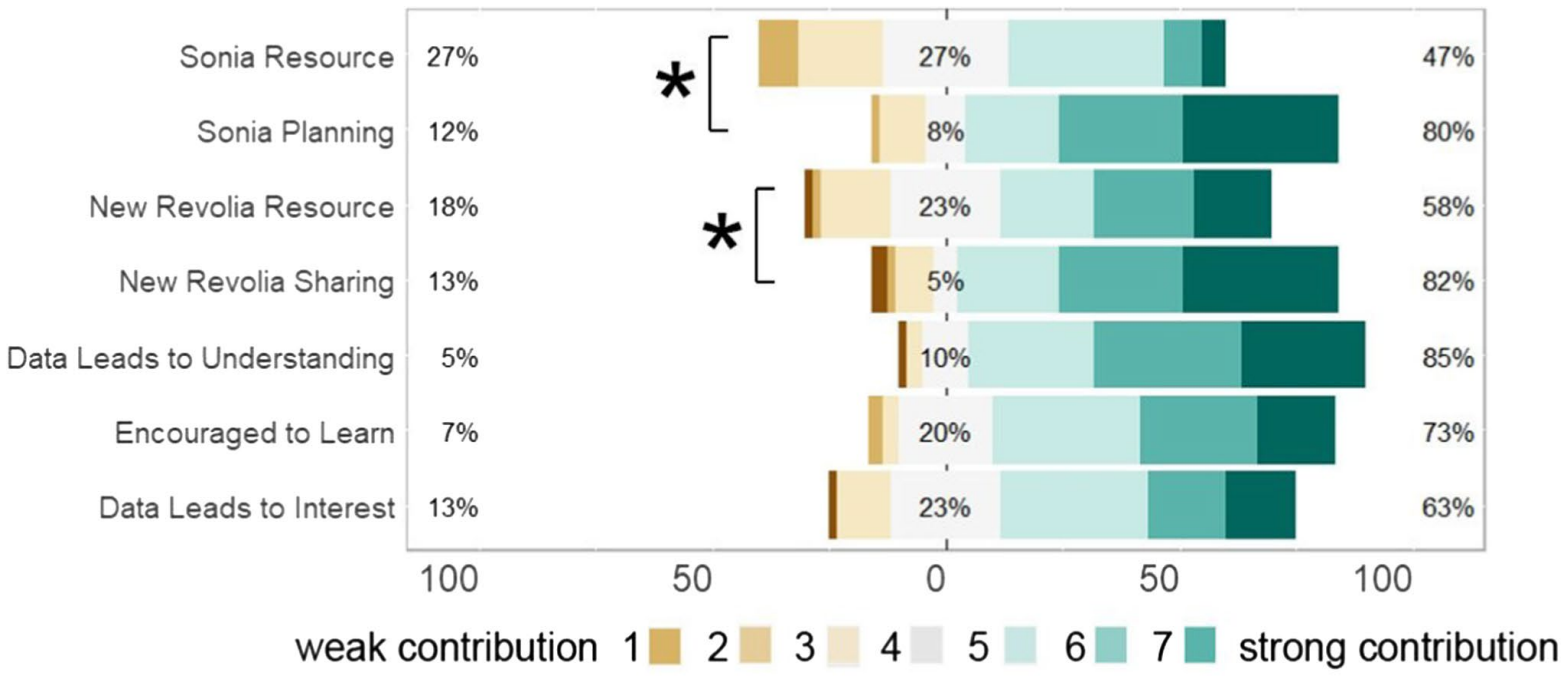
Quantitative results from survey on 1–7 Likert scale. Sonia.Resource—“How strongly does the Sonia McDougal story contribute to an understanding of development of resources?” Sonia.Planning—“How strongly does the Sonia McDougal story contribute to an understanding of prioritizing long term planning?” New.Revolia.Resource—“How strongly does the New Revolia story contribute to an understanding of resource limitations?” New.Revolia.Sharing—“How strongly does the New Revolia story contribute to an understanding of sharing amongst diverse groups?” Data.Leads.to.Understanding—“How strongly do the four panels with specific climate change data and facts contribute to your realization of the urgent situation of climate change?” Encouraged.to.Learn—“How encouraged are you by the data shown in the manga pages to learn about climate change?” Data.Leads.to.Interest—“How strongly do you think this data in comics contributes to your interest in climate change evidence?” Sonia Resource responses differ significantly from Sonia Planning; New Revolia Resource responses differ significantly from New Revolia Sharing (Wilcoxon ranked sum, *p* < 0.05).

In general, participants reported being quite positive about the way the design of stories and data visuals contributed to learning about climate change (Data Leads to Understanding, Encouraged To Learn, Data Leads to Interest in Figure 7). In the Sonia McDougal story, there is a significant difference between the way people perceived the climate change purpose (median 6, mean 5.6) as opposed to the edification over physical resource level (median 4, mean 4.3; Wilcoxon ranked sums, *p* = 6.15*e*–7), showing evidence that the story design led readers to consider the idea of future-directed thinking (the core theme of the story) more than the physical aspects of climate change. In the New Revolia story, there was a significantly higher understanding of the theme of resource sharing (median 6, mean 5.53) over understanding of resource limitation (median 5, mean 4.9) resulting from reading the story (Wilcoxon ranked sums, *p* = 0.008902), consistent with the view that the story design led to better understanding of the human theme of sharing as opposed to teaching about climate change on the physical level.

**Figure 8:**
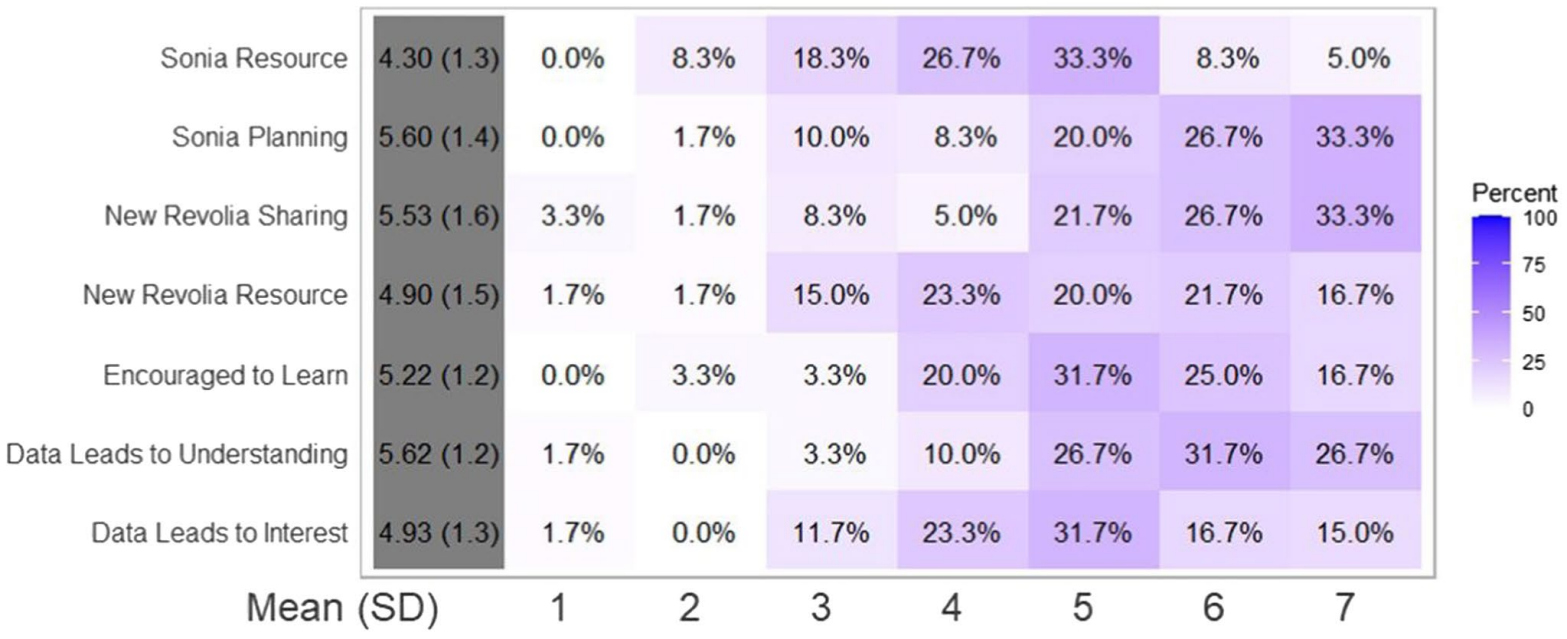
Quantitative results from survey on 1–7 Likert scale shown as heatmap to show the variation in the data. Questions same as in Figure 6.

The Likert data is shown in Figure 7, with the variability of the data shown in the heatmap in Figure 8. Note that variability in the response to each question set are similar (interquartile ranges in Sonia Resource, Sonia Planning, New Revolia Sharing, and New Revolia Resource are all 2; variances in the respective groups are 1.57, 1.91, 2.16, and 2.42, Table 1; Sonia Resource vs. Sonia Planning *F*-test; *p* = 0.8238; New Revolia Sharing vs. New Revolia Resource, *F*-test; *p* = 0.8913).

**Table 1:** Descriptive statistics for Likert scores for each survey question as asked in Figure 6.

Survey question	Mean	Median	Variance	IQR
Sonia Resource	4.30	4	1.57	2
Sonia Planning	5.60	6	1.91	2
New Revolia Resource	4.90	5	2.16	2
New Revolia Sharing	5.53	6	2.42	2
Data Leads to Understanding	5.62	6	1.53	2
Encouraged to Learn	5.22	5	1.53	2
Data Leads to Interest	4.93	5	1.76	2

The positivity about climate change can be due to the self-reported support of climate change, for the climate change attitude scale revealed a general agreement of over 80% above 4 (1–7 scale) in every question in the survey, e.g., “How strongly do you believe the facts and data of climate change?” “How strongly do you agree climate change will impact future generations?” (Christensen and Knezek, 2015). This indicates that readers already report highly on pro-climate attitudes, and hence the story may not be tested against a population that are climate change skeptics. Indeed as seen in Figure 6, the number of participants over 50 in our study was low, reflecting the relative lack of anti-climate change viewpoints in our study.

## Results: Qualitative

*Reactions to data comics:* When asking about the story based questions (including individual reactions to specific story plots, graphs, the decisions of characters, future development of stories etc.), participants obtained a better understanding based on visual attributes of the data (including visual symbols, colors, shapes and sizes). In particular, they mentioned the benefits of data visuals for adding credibility and acceptability to narrations in comics.

“*The smaller you are, the less you give and should take.*”—P3

“*The graph is simple, but it adds credibility to the comic.*”—P14

*“Food given based on how much each person will contribute to CO2 emission, so they are trying to minimize emission.”*—P17

“*Easy to understand. The shapes of the circles are in different sizes depending on the person, and that's the amount of resources they should get in "box quantities*.”—P18

“*The first graph supports Sonia's statement that weather has been unpredictable, but also shows a level increase in the amount of rainfall. The second image shows how the weather patterns shown in the first image have developed into storms.*”—P44

However, some participants may reconsider the rationality of stories based on the accuracy of data shown in comics. Thus, designers should be more cautious in selecting and presenting reliable and scientific data in the storytelling context if they are used to persuading traditional argument strategies. For instance, several responses queried the fairness of the distribution plan shown in the graph based on personal understanding and reflection.

“*The figures don't involve any specific gender.*”—P36

“*They use pseudo science environmental charts*.”—P33

“*Not very scientific.*”—P44

These comments show that readers understand the data parts of the comic are not scientific, but are designed for the narratives of the story. Thus the data visual is deemed fictional, since they emanate from hypothetical scenarios that extrapolate based on current climate change understanding.

*Personal perceptions about climate action purpose:* Responses from participants who already have good climate awareness suggested that data comics is a more engaging and provoking form for viewers (P44: “*I am already well informed on the subject but it definitely engaged my interest and I found it very thought provoking*”). However, several participants mentioned that data may become exaggerated and fictitious during the process of visualization (P6: “*The data in the comics is more fictional than the scientific data*”; P8: “*The comic maybe present some exaggeration due to impact in the viewer, and also add some color and shapes*”). Although participants may feel confused about the accuracy of data in comics and fictional stories, they still preferred data comics as a creative climate change communication (P14: “*The data from the comic is very generalized and imprecise, but it is expressive and attracts the reader’s attention*”).

*Limitations of data comics:* Based on the analysis of participants’ willingness to adopt pro-climate change actions, a limitation of data comics was the length of stories. Participants emphasized the significance of the length of story lines which can make audiences immersive in the story’s environment.

*“The comics were too short to provoke a catarsis.” -*P16

" … *longer story lines to take one on a journey*." -P10

On the other hand, participants also pointed out that they realized the urgency of climate change not only from reading these climate action comics (P36: “*Not from the comic alone*”). To further support the effectiveness of data visuals in climate comics, designers should think more about the connections between narrations and specific situations in reality which may enable users to take pro-climate change behaviors (P14: “*It is important to show specific situations with which the reader can identify and imagine himself in the future*”).

## Discussion

To verify the validity of data visuals in combination with design fiction for the implicit influence of climate change communication, we explored how speculative data comics can be designed to enable climate change awareness and potentially nudge audiences to take pro-environmental behaviors in a visually appealing and intelligible way. We designed and distributed a comic magazine that has the covert purpose of climate action without advertising itself as about climate change, the narrative contents of the comics are designed for positive persuasion rather than showing negative facts. We then evaluated the way the comic stories which presented human phenomena can inform readers in regards to goals and motivations as opposed to resource and policy data. Quantitative data showed that participants were more likely to focus on the theme of future-thinking in a story as opposed to physical issues like resource limitations. In the qualitative data, several participants felt confused about the rationality and accuracy of data comics and fictional stories, highlighting the understanding of the narrative component of the story rather than the scientific data. Although the findings cannot support that climate storytelling is more persuasive than communicating evidence, it shows comics are more attractive and understandable. This provides implications for designing stories in the future: providing implicit cues for understanding alignment with climate action rather than explicit arguments as they appear to be more receptive by audiences. In general, readers reported positive influences in climate awareness and alignment with positive climate action goals.

However, regarding the majority of our participants reported having a pro-environment perspective, our study demonstrates that speculative data comics let people with pre-existing environmental attitudes have more possibilities to activate pro-environmental behavior. This suggests that to better evaluate our design, we should look for groups more antagonistic and from more nations to climate action to better evaluate the implicit effects of narratives and speculative data on climate change attitudes, because government policies, green economy levels and climate change educational efforts in different countries still impact the formation of public attitudes about environmental issues. These further insights would provide better design strategies for both data visuals and story design for particularly needed groups to change pre-existing attitudes before they are fixed completely such as climate change deniers.

## Conclusion

Speculative data comics with positive and implicit messages have the potential for reducing the sense of distance between climate change and the public. Comics are a more accessible, understable, and memorable way of climate communication for the general public. Our work introduced a novel design strategy directed at influencing the audience’s motivations and goals in alignment with climate action, as opposed to education in regards to the facts in climate issues. We recognize the difficulty in making long-term changes in opinion, and the even greater difficulty in measuring the long-term effects of these changes. However, we believe that design strategies for climate communication exemplified here provide an implicit influence that may, in the long run, have an impact on human perceptions and actions in the climate change debate.

## Data availability statement

The raw data supporting the conclusions of this article will be made available by the authors, without undue reservation.

## Ethics statement

The studies involving human participants were reviewed and approved by City University of Hong Kong. The patients/participants provided their written informed consent to participate in this study.

## Author contributions

RL, ZS, and YS created the stories and the drawings. RL, CY, and ZS created the figures. RL, CY, YS, and ZS wrote the manuscript. All authors contributed to the article and approved the submitted version.

## Funding

The research has been partly supported by the Kyoto Design Lab Researcher in Residence program.

## Conflict of interest

The authors declare that the research was conducted in the absence of any commercial or financial relationships that could be construed as a potential conflict of interest.

## Publisher’s note

All claims expressed in this article are solely those of the authors and do not necessarily represent those of their affiliated organizations, or those of the publisher, the editors and the reviewers. Any product that may be evaluated in this article, or claim that may be made by its manufacturer, is not guaranteed or endorsed by the publisher.
